# Brief report: Serial capillary lactate measurement predict the evolution of early sepsis

**Published:** 2016

**Authors:** A Purcarea, A Bourgarit, A Sovaila, C Ghiura, P Diemunsch, E Andres

**Affiliations:** * Internal Medicine, “Hopital Civil” Hospital, Strasbourg, France; ** Internal Medicine Department, Internist.ro, Brasov, Romania; *** Internal Medicine, “Hautepierre” Hospital, Strasbourg, France; **** Anesthesiology, Critical Care and Prehospital Emergency Medicine, Strasbourg, France

**Keywords:** sepsis, capillary lactate, outcome prediction

## Abstract

Objective: In intensive care settings, blood lactate level measurement proved to be an excellent predictor of outcomes. In patients requiring less urgent treatment, the arterial blood lactate is less sensitive and its usefulness remains to be proven. Capillary blood lactate dosing, an emergent point-of-care technique readily available should be more sensitive to changes in these settings.

Method: prospective, observational, monocentric study conducted in a polyvalent internal medicine ward in a French University Hospital. The inclusion criteria were the existence of new symptoms of abrupt onset in an otherwise stable patient. All the patients had a point of care measurement of baseline capillary and venous lactate levels (EDGE, ApexBio) and standardized control before any therapeutic means were initiated. A follow-up test was performed once again within 12 to 36 hours. All the patients received standard medical care adapted to their condition. The primary outcomes were considered dying within 30 days or requiring intensive care or invasive therapeutic procedures.

Results: 13 patients were analyzed. Seven patients reached the composite outcome with 3 deaths. The superimposed complication proved to be infectious in every case. The median lactate levels were at baseline (mmol/ l): capillary Mc0=5.2(2.16), venous Mv0=2.3(2.0) and arterial Ma0=1.8(1.7) and at follow-up (mmol/ l) capillary: Mc1=3.3(1.1), venous Mv1=1.8(1.8) and arterial Ma1=1.3(0.7).

In nonparametric analysis, the absence of normalization of capillary lactate at follow-up was correlated well with poor outcomes (p=.05). This was not the case of arterial or venous lactate measurements. The positive lactate clearance was present in the majority of patients (83.3%) but it did not predict the outcomes (p=.435) and there was no correlation between the baseline lactate and the clinical outcome (p>.05).

Conclusion: In non intensive care settings, capillary lactate level could be a more sensitive method than the classical lactate measurement for predicting the outcomes of acute conditions, especially infectious. A persistently high lactate level rather than its initial value or clearance seems to correlate better with poorer outcomes.

Abbreviations: SSC = Surviving sepsis campaign, ED = Emergency department, ICU = intensive care unit, , POC = Point of care, ICC = inter class coefficient

## Introduction

Infection, sepsis, severe sepsis and septic shock represent a continuum of diseases [**1**], which impose an important human and economic burden [**2**,**3**,**4**,**5**]. The arrival of the “Surviving sepsis campaign” (SSC) represented a significant breakthrough in the early control of severe sepsis and septic shock [**6**]. It greatly improved the survival rates in the emergency departments and intensive care units for patients already suffering from complicated sepsis, however, limitations still exist, especially outside the intensive care ward [**7**,**8**,**9**]. It should be noticed that over 50% of all severe sepsis and septic shock cases would occur outside the ED or ICU [**7**]. At least in part, this is due to a lack of sensitive diagnostic tools that would allow the identification and limitation of the natural progression of severe infections from sepsis to septic shock especially in the current frail inpatient population [**10**,**11**]. There is a need for a new or better use of already available biomarkers and clinical scores that could accelerate the diagnosis [**12**,**13**].

Serum lactic acid level and lactacidemia are valid witnesses of the perfusion and metabolic changes related to sepsis and the increase in oxygen needs. Lactacidemia correlates well with sepsis severity and makes proof of a prognostic value even at intermediate levels [**14**,**15**,**16**], therefore being included in the international treatment guidelines for severe sepsis and septic shock.

The point of care capillary lactate is an alternative method of blood lactate measurement already tested and accepted in sepsis management. However, it seems to overestimate the “classical” arterial measurement in severely ill patients with overt tissue hypo perfusion but has never been tested in the early stages of sepsis when it could be a more accurate witness of an ongoing occult process [**17**,**18**].

In order to evaluate the accuracy of the outcome prediction of point-of-care capillary lactate measurements in a population of patients with suspected ongoing sepsis, we presented the results of a pilot study that compared the point-of-care capillary lactate to venous and arterial measurements.

## Method

### Patients

All the patients with a suspected new onset sepsis were prospectively included, over 6 months in late 2012 and early 2013 in the Acute Medicine Unit of an Internal Medicine Department of a tertiary care hospital in Strasbourg, France.

The required inclusion criterion was the existence of a new superimposed clinical sign of sepsis of abrupt onset (polypnea, tachycardia, fever, or chills) in an otherwise stable patient hospitalized for an acute or chronic condition.

The exclusion criteria included the age of less than 18 years, the initiation of therapeutic maneuvers more than 15 minutes before blood samples were obtained, a medical condition with a life expectancy of less than 1 month, the necessity of intravenous or inhaled beta-mimetic bronchodilators and a low initial blood lactate level (arterial, venous and capillary).

Patients were followed up until death or hospital discharge in order to provide the 1-month mortality or invasive treatment data.

This pilot study was discussed with the local ethics committee and the explorations were considered part of the standard clinical practice (articles L.1121-1 paragraph 1 and R1121-2, French Public Health Code).

### Outcome measurement

The composite primary outcome included dying within 30 days, or requiring admission in an ICU for shock or invasive therapeutic procedures. The transfer to a critical care unit was decided if a diagnosis of septic shock was considered. The absence of a significant clinical and biological response to the initial treatment within 48 to 72 hours was considered on grounds of invasive procedures if such a procedure was deemed appropriate. 

### Lactate measurement

Blood samples that were analyzed in the central laboratory included arterial (radial) lactate and other serum parameters (venous), as encouraged by the current policy in such cases. Fingertip point-of-care capillary glucose measurements were also performed on a regular basis in diabetic and non-diabetic patients with suspected sepsis. At that same moment, point of care measurements of baseline capillary (fingertip) and venous (arm) lactate levels (EDGE system by ApexBio©, Taiwan) were performed. This device was approved for POC testing of lactate in Europe, has a detection range of 0.7 to 22.2 mmol/ l and uses single-use test strips containing an enzyme coated electrode. The machine was calibrated by the study investigator and was tested each day with a check-strip with a known value. Patients received finger pricks for glucose control with a disposable lancet and the second capillary blood drop was used to obtain the capillary lactate level.

For patients with arterial, capillary or venous lactate levels superior to the central laboratory accepted normal values (2.2 mmol/ l), we considered it justified to perform the test once again no sooner than 6 hours and not later than 72. The standard-of-care non-invasive measures specific to each patient’s initial state were enacted, regardless of the lactate level. Treating physicians were not blinded to either POC or the laboratory lactate values. 

### Lactate measurement

Blood samples that were analyzed in the central laboratory included arterial (radial) lactate and other serum parameters (venous), as encouraged by the current policy in such cases. Fingertip point-of-care capillary glucose measurements were also performed on a regular basis in diabetic and non-diabetic patients with suspected sepsis. At that same moment, point of care measurements of baseline capillary (fingertip) and venous (arm) lactate levels (EDGE system by ApexBio©, Taiwan) were performed. This device was approved for POC testing of lactate in Europe, has a detection range of 0.7 to 22.2 mmol/ l and uses single-use test strips containing an enzyme coated electrode. The machine was calibrated by the study investigator and was tested each day with a check-strip with a known value. Patients received finger pricks for glucose control with a disposable lancet and the second capillary blood drop was used to obtain the capillary lactate level.

For patients with arterial, capillary or venous lactate levels superior to the central laboratory accepted normal values (2.2 mmol/ l), we considered it justified to perform the test once again no sooner than 6 hours and not later than 72. The standard-of-care non-invasive measures specific to each patient’s initial state were enacted, regardless of the lactate level. Treating physicians were not blinded to either POC or the laboratory lactate values. 

### Definitions

Suspected sepsis was defined as the presence of a possible infection site and the presence of one clinical sign of systemic inflammatory response syndrome with no other possible explanation. As others did, we used the consensual definition of sepsis: it represents a suspected or proven infection in the presence of two or more clinical or biological systemic inflammatory response syndrome criteria. Severe sepsis was defined as sepsis associated with acute organ dysfunction or hypotension by the same international consensus and septic shock was defined as sepsis with acute circulatory failure characterized by persistent arterial hypotension (systolic arterial pressure <90 mmHg, mean arterial pressure <60 mmHg, or a reduction in systolic blood pressure >40 mmHg from baseline) despite the adequate fluid challenge [**6**,**19**,**20**]. 

Initial arterial, venous or capillary lactate levels were categorized as low (<2.2 mmol/ l), intermediate (>2.2 and <4.0 mmol/ l), or high (>4 mmol/ l).

Lactate clearance was defined according to the formula : (Lbaseline - Lfollow-up)/(Lbaseline ) x 100 [**21**]. A positive lactate clearance is considered if the calculated value is superior to 10%.

### Data-collection

The following variables were considered as potential confounders: demographics, comorbidity, previous medical history, vital signs, suspected infection site, laboratory data, time variables, and treatment variables. Data on each of these factors was collected in the patient’s medical record.

### Statistical analysis

Data was presented as median (M) and inter quartile range (IQR). It was verified for normality with the Shapiro test and it was found to be nonparametric. The statistical variance between groups was calculated with the Mann-Whitney U test. The strength of correlation with the primary outcome was assessed by using the Pearson’s r correlation coefficient. The agreement between measurements was assessed with the ICC. ICC values of over .6 were considered statistically significant, values over .8 were considered good and over .9 were considered excellent. The statistical analysis was performed by using SPSS for Windows release 22, Chicago, Illinois, and p<.05 was considered significant.

## Results

### Baseline characteristics

16 already hospitalized patients met newly suspected sepsis criteria during the study period. Of these, a total of 13 patients were kept for follow-up. Among the 3 excluded patients, 2 had normal lactate levels (any site) and one received bronchodilators minutes before the blood sample was drawn. Eight of the 13 analyzable patients were men (61.5%) with a median age of 75. Initially suspected sepsis was confirmed for all 13 patients. The infectious sites were: pulmonary (n=9) (2 abscesses), digestive (n=1), osteomyelitis (n=1), endocarditis (n=1), urinary (n=1) (**Table 1**). The median Charlson Comorbidity Index was 6 (3-13). Seven patients (53%) reached the primary outcome whereas 6 recovered and were sub sequentially discharged after the onsite treatment. Of the 7 patients who reached the primary outcome, 3 patients died within the first 28 days.

**Table 1:**
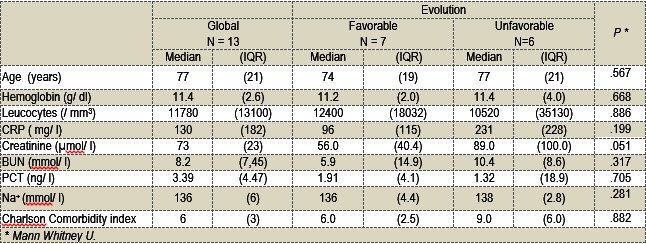
**Table 1**Baseline demographics. CRP = C reactive protein, BUN = blood urea nitrogen, PCT = procalcitonin, Charlson = Age adjusted Charlson’s comorbidity index

### Lactate measurements

Median lactate levels for all 13 patients were (mmol/ l): at baseline - capillary Mc0=5.2 (2.16), venous Mv0=2.3 (2.0) and arterial Ma0=1.8 (1.7) and at follow-up (mmol/ l) - capillary: Mc1=3.3 (1.1), venous Mv1=2.2 (1.6) and arterial Ma1=1.3 (0.7) (**Table 2**). Lactate clearance was positive, with a median Mla = +5.4% (64%). Venous and capillary lactate clearance median values were respectively Mlv = +16% (49%) and Mlc = + 39% (22.5%).

**Table 2:**
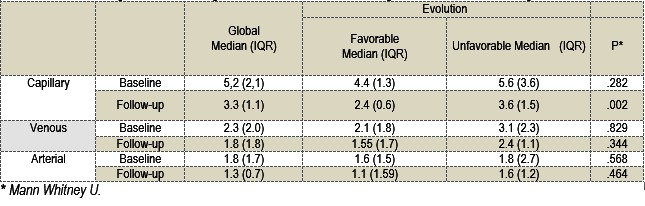
**Table 1**Lactate levels, global and according to the evolution with a statistical significance and correlation degree

 The agreement between venous and arterial lactate samples was excellent at baseline and good at follow-up (ICC of .909 at baseline and .894 at follow-up). The capillary lactate values did not agree with either venous or arterial lactate (ICC < .6). Lactate clearances (capillary, venous, and arterial) were in good agreement with the 3 tested methods (ICC .732).

**Fig. 1:**
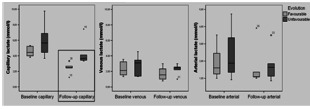
**Fig. 1**Differences between lactate levels according to measurement types (capillary, venous, and arterial) at baseline and follow-up for patients with a favorable evolution versus patients who met the primary outcome. The statistical significance is expressed by the Mann Whitney test (**Table 2**)

There were no significant differences between the patients with or without poor outcomes at baseline (**Table 2**). The median lactate levels of the two groups at follow-up were (mmol/ l): capillary M1c = 2.4 (0.6) vs. 3.6 (1.5), venous M1v: 1.55 (1.7) vs. 2.4 (1.1), arterial M1a: 1.1 (1.59) vs. 1.6 (1.2) (**Table 2**). The capillary lactate value (M1c) distinguished patients according to the primary outcome (Mann Whitney Z: -2.817, p=0.005 with Pearson’s r = 0.637; p=0.026). Arterial and venous samples did not reach the statistical significance for the primary outcome (**Table 2, Fig. 1**). For a cut-off value of 2.6 mmol/ l the ROC Curve analysis identified a sensitivity of 100% and a specificity of 83,3% for the follow-up capillary lactate, whereas, a 3,38 mmol/ l cut-off had a 100% specificity and a 83,3% sensitivity.

## Discussion

This monocentric pilot study gave evidence that in early stages of sepsis, capillary lactate was a better biomarker of evolution than the venous or arterial levels, especially if performed in a seriate way.

Sepsis can set in and evolve silently to severe states in the internal medicine ward patients, while the initial process is masked by comorbidity or concomitant medications in a mostly frail population [**7**]. For example, all our patients were hospitalized before the onset of the septic episode and were at least partially treated. Only half of them ever presented with fever. In these peculiar settings, the probable reasons for the rise of sepsis mortality after the SSC guidelines implementation were that the initial treatment would not always perfectly control the evolution from infection to septic shock. Anticipating with appropriate biomarkers the re-emergence of sepsis in this frail and ageing population with limited immune responses to aggression should be one of the best targets in order to control the rise in mortality [**10**].

Although consecrated as valuable sepsis biomarkers, in our study, the arterial and venous lactate, at either baseline or follow up, did not reach a statistical significance, showing only trends for the primary outcome. Their median value was an intermediary one. We hypothesized that the lack of power of our study explained the difference to other evidences where arterial lactate, even at intermediary levels proved some prognostic value for sepsis evolution [**16**,**28**].

The venous levels controlled with the POC device and arterial lactate levels determent in central laboratory were in excellent agreement [**25**,**26**]. This should have also excluded a potential bias due to the test methods.

At baseline, alongside arterial and venous, capillary lactate had no clear prognostic value for the primary outcome but showed the same clear trend for the differentiation between the patient’s evolution.

At follow-up, the capillary lactate value proved to be a good biomarker for the ongoing sepsis and outperformed arterial and venous measurements. The hemodynamic and vasomotor changes in the early or compensated sepsis initially occurred peripherally and probably continued if the process was not completely controlled, with a persistent microcirculatory dysfunction. This would lead to an excess local anaerobic metabolism and lactate production even after the achievement of normal global oxygen delivery and normalization of arterial lactate [**14**,**22**]. For this specific state, the fingertip and the earlobe capillary lactate should outperform arterial and venous samples. We chose the fingertip and not the earlobe capillary measurement because the former could be considered a lot closer to the whole blood values. Recent data confirms our hypothesis [**24**]. Also, in conditions others than sepsis, evidence supports the existence of different lactate levels for different sampling sites [**27**].

As previously defined, the minimum required lactate clearance was achieved for the majority of our patients, without any prognostic significance in our case. This is explained by the persistently abnormal high values at follow-up even when a clearance of 10% was achieved. This comes in accordance with recent findings, that not being the downwards trend but the final lactate value which had the greatest prognostic value [**29**,**30**].

The previous studies on lactate in sepsis were centered on mortality in the ICU of severe sepsis or septic shock, ours being on an early intervention in order to prevent it [**6**,**17**,**23**]. Although with optimistic results, our open, monocentric, small sample size, observational pilot study had several limitations and needs to be prospectively confirmed (Clinicaltrials.gov number: NCT02180399). The exact time and cut-off value should be determined in larger studies. When sepsis is suspected, we suggest that serial systematic capillary lactate measurements would yield the best results.

## Conclusion

In non intensive care settings, capillary lactate level could be a more sensitive method than classical lactate measurement for the outcome prediction of acute infectious conditions. A persistently high capillary lactate level over a proposed value of 2,6 to 3,4 mmol/ l after the initial intervention, seems to correlate better with poorer outcomes.
